# An Integrated Multi-Objective Optimization for Dynamic Airport Shuttle Bus Location, Route Design and Departure Frequency Setting Problem

**DOI:** 10.3390/ijerph192114469

**Published:** 2022-11-04

**Authors:** Ming Wei, Congxin Yang, Tao Liu

**Affiliations:** 1 School of Air Traffic Management, Civil Aviation University of China, Tianjin 300300, China; 2 School of Transportation, Nantong University, Nantong 226019, China; 3 National Engineering Laboratory of Integrated Transportation Big Data Application Technology, School of Transportation and Logistics, Southwest Jiaotong University, Chengdu 611756, China

**Keywords:** airport shuttle bus, route design, departure frequency, multi-objective mixed-integer linear programming, Pareto-optimal solutions, time-varying demand, station selection

## Abstract

An airport shuttle bus (ASB), as an environmentally friendly mode of green transportation, is an effective way to solve the “first/last mile” of aviation passengers, which can attract a higher passenger transfer from private cars to public transport, thereby reducing emissions of carbon dioxide and other polluting gases. This study presents a multi-objective mixed-integer linear programming for ASB services in a dynamic environment. Taking into account time-varying demand and travel time characteristics in different periods, the proposed model provides a comprehensive framework that simultaneously advises passengers to join the bus at the nearest bus stations, designs routes for transporting them from these selected stations through the airport, and computes their departure frequencies in multiple periods. The primary objective is to optimize both the total ride time and waiting time for all passengers. The secondary objective is to optimize the total transfer distance of all passengers simultaneously. Given the Non-Deterministic Polynomial (NP) hardness of this problem, a two-stage multi-objective heuristic approach based on the non-dominated sorting genetic algorithm (NSGA-II) is combined with a dynamic programming search method and further advanced to obtain the Pareto-optimal solutions of the proposed model within a reasonable time. Finally, the proposed model and algorithm feasibility are proved by a test example of designing a shuttle bus route and schedule at Tianjin Airport, China. The results show that the total passenger travel time of the presented model is markedly reduced by 1.21% compared with the conventional model.

## 1. Introduction

**Figure 1:**
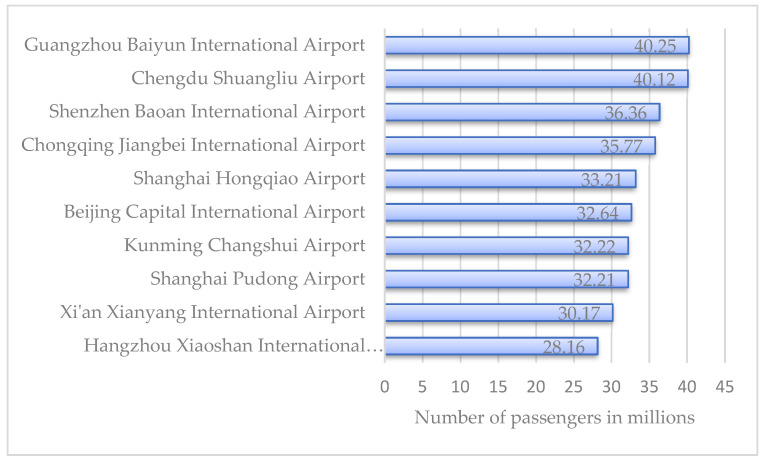
Top-ten leading airports in mainland China in 2021, by passenger throughput.

Owing to the economic growth worldwide, air travel demand has grown significantly [1]; thus, demand for air travel has more than doubled between 2005 and 2021. This has yielded a significant amount of traffic and passenger flows to the airport. As shown in Figure 1, the passenger throughput in Guangzhou Baiyun international airport has reached 40 million. The increasing demand on air traffic has resulted in a growing concern in ground access to the airport [2]. Similar to the conventional bus, the airport shuttle bus is the most important mode of transportation linking the airport and the city when the airport rail transit has not been widely used [3].

An airport shuttle bus (ASB), as an environmentally friendly mode of green transportation, is an effective way to solve the “first/last mile” of aviation passengers, which can evacuate all of them from large suburban and remote residential areas to the airport. Compared with private cars, new-energy ASBs have the advantages of zero emission, no pollution, low noise, and little energy consumption. Hence, the development of ASB services is beneficial to promote energy conservation and emission reduction, which could reduce energy consumption, actively cope with the global energy shortage, and improve urban air quality [1,4,5,6]. The ASB network (ASBN), which consists of a group of nodes (airport, bus stations, demand points, and dispatch centers), as well as the connections among them, aims to design several routes to visit these nodes to cover all demand points and optimize their departure frequencies, which is directly related to passenger travel convenience, scheduling efficiencies of routes, and carbon emissions of buses. Therefore, authorities must find a well-designed airport shuttle bus network to reduce operational costs and carbon emissions and improve mobility.

Route planning and frequency setting are two major tasks in the airport shuttle bus network (ASBN), similar to the conventional bus network (CBN). The former provides the data for the latter, and the two affect each other. Numerous studies have investigated the implications of frequencies in diverse route configurations on satisfying the identical demand of passengers. Nevertheless, most ASBN/CBN designs fail to establish bus route configurations or set frequencies as a combined operation [7,8,9]. In addition, most researchers assumed that bus stations were where demand points were located and that they were all visited. Passengers can walk or transfer to a close station to board and disembark the bus at the demand point in the ASBN, and their preferences are impacted by available seats and less ride time on the route. Designing ASBN routes and frequencies also requires selecting the best stations in alternative shuttle locations [10,11]. Hence, it is vital to find a way to integrate route design and frequency determination with station choice for ASB operations to enhance service level and operational benefits.

Because passenger flow and travel time are the primary data for designing an ASBN, most scholars have studied this problem with static demand and travel time during peak hours, which could only design all routes and optimize their departure frequencies in a single period. However, few take time-varying demand and travel time into account. Travel demand and time vary from time to time, which fluctuates with regularity. In this case, an ASBN in a static environment cannot deal with such a dynamic one. Generally, the day can be divided into several periods, including morning and evening peaks and low peak and peak hours. An ASBN that considers the multiperiod travel demand/time is more scientific and reasonable than a single-period ASBN but is much more complicated. Hence, it is essential to reveal the influence of multi-period travel demand/time changes on integrated ASB operations.

As stated above, few investigated the integrated operation of route design and frequency determination with station choice for ASB, and research focusing on optimizing the frequency determination and the transit route design with station choice concurrently in a dynamic environment is even sparser. The primary goal of this research was to explore a comprehensive multi-objective optimization framework to balance the total transfer distance between demand points and shuttle stations, the total waiting time at shuttle stations, and the total ride time between bus stations and airports for all passengers. This framework coordinates passenger boarding guidance, bus station selection, route design, and frequency-setting procedures in multiple periods.

The remaining study is shown as follows: The related work on ASBNs is summarized in Section 2. The methodology of the issue and a mathematical formula for the comprehensive model are proposed in Section 3. A two-phase multi-objective NSGA-II-based solution to address this issue is proposed in Section 4. A case study is presented in this research, followed by comparative and sensitivity analyses in Section 5. Finally, the conclusions and directions of future research are summarized in the final section.

## 2. Related Work

The airport shuttle bus (ASB) is the most critical mode of transportation linking the airport and the city when airport rail transit has not been widely used. The reasonable design of ASB services is directly related to the convenience and rapidity of travelers transported from bus stations to airports, affecting air transport quality and efficiency. Regarding ASB operating costs, in-vehicle congestion, ride time, station waiting time, route design, and frequency determination are essential elements to enhance the quality of service [12]. Most prior research focused on frequency optimization to provide a timetable based on an available ASB route network. The operational productivity of an ASB system cannot be maximized by the integration of transit route architecture and service frequencies. Therefore, it is crucial to optimize the frequency determination and the transit route design concurrently.

Interest in integrating ASBN/CBN route design and frequency determinations has grown recently. Sequential methods, transit route design considerations into frequency determination, or the inverse did not always result in a globally optimal solution in early research investigations [13]. The following methods were utilized to manage the integration operation to avoid these drawbacks: (1) partial integration, i.e., bus route design considering frequency determination [14], and (2) full integrations, i.e., coherent decision-making for both issues [15]. Decisions related to each subproblem handled by fully integrated operations are substantially more complex than the subproblem. [16]. Additionally, various precise and heuristic methods for handling complicated issues have been provided [9,17]. The primary research tasks are listed below:

The bus routes and frequency determinations were established for the bi-level bus network configuration problem study [8]. The lower-level problem was the transit allocation problem with volume limitations. The goal of the upper-level problem, which consisted of mixed-integer nonlinear programming, was to reduce the number of passenger transfers. To increase transportation hub connectivity and service quality, Wei et al. [12] proposed a mathematical model with an integrated framework to create a shuttle transit service. Ruano-Daza et al. [13] proposed a multi-objective, two-level strategy based on globally optimized search algorithms to find the optimal BRT system routes and frequency. To integrate the collection of feeder routes as well as its corresponding serving frequency to minimize customer expense and unsatisfied demand, Buba and Lee [15] presented a differential evolution method. To find solutions that benefit both passengers and operators, Ahern et al. [17] focused on the integrated optimization of route network plans and the departure frequency determination integration of bus transport systems. In railroad rapid transport networks, López-Ramos et al. [18] introduced a comprehensive optimization approach to address both the network design and frequency determination phases. Sun et al. [19] presented a transfer optimization model for the county bus network to deal with the problem of improper bus network planning, inconvenient operation optimizations, and insufficient protection measures in the country bus network system. Shang et al. [20] solved an integrated operational problem regarding hierarchical service network design and passenger assignment for urban rail transit systems. Zhou et al. [21] considered a line planning problem in an urban rail transit (URT) network with passenger path assignment. Wu et al. [22] presented an integrated optimization model incorporating ridership matching and shuttle scheduling into one framework. Bus trip scheduling and passenger–vehicle matching schemes were optimized. Sigler et al. [23] added to the limited literature on airport shuttle route optimization with customer satisfaction, and energy reduction was handled simultaneously.

## 3. Research Gaps and Contributions

As illustrated in Table 1, the most frequent objectives are the integrated ASBN/CBN optimization model’s operational expenses and passenger assessment metrics. A few exact algorithms can only resolve small-scale cases quickly. For large-scale cases such as airport shuttle bus route design, advanced metaheuristic algorithms are generally used to solve the problem. In addition, ASBN/CBN routes are designed to primarily address the static travel time, operating costs, and network density of passengers, with little regard for the dynamic travel demand and time.

The literature review on ASBN/CBN identified the three key issues that require a more profound exploration:

1. Although some studies have performed a synergistic optimization of route design and frequency determination in CBNs, these factors have rarely been considered in ASBNs. Due to the disparity between demand patterns and route architectures when people at demand points can walk or transfer to one of the nearest stations to get on and off the ASB, in comparison to the conventional integration model, the comprehensive operation of route design and frequency determination with the allocation of each demand point to the selected station was more effective [24].
2. Most researchers have neglected time-varying travel demand in ASBNs and only made routing and scheduling strategies in a single period. Few studies have studied ASBNs considering multiperiod travel demand in a dynamic environment [25,26].
3. Such an integrated ASBN is a multi-objective NP-hard problem, and an effective heuristic method must be utilized to create a group of Pareto solutions to determine the ideal operational cost-to-convenience ratio for bus companies.

According to the gaps in the literature, the main contributions of this research are summarized as follows:

- Coordination of ASBN transit location, routing, and scheduling process in a dynamic environment to balance passenger and company benefits;
- Development of a two-phase multi-objective heuristic approach based on the non-dominated sorting genetic algorithm (NSGA-II) to acquire a group of Pareto-optimal solutions efficiently;
- Finally, an exact instance is used to demonstrate the effectiveness and practicality of this study.

## 4. Methodology

### 4.1. Research Framework

The key inputs to this study were the travel demand of demand points and the travel distance/time matrix between demand points, bus stations, dispatch centers, and the airport in different periods. This study presented a multi-objective integrated optimal model for an ASBN service to coordinate the station location, route design, and frequency determination process in a dynamic environment, thus guiding passengers to walk or transfer from demand points to selected stations and transporting them from these stations to the airport by ASB. In the locating and routing model, each ASBN route departed from the dispatch center, visited some stations, and arrived at the airport, where only one selected station could be allocated to a demand point. The station was able to be assigned to more than one demand point. Some constraints, such as the capacity of the station and route, maximum walk or transfer distance, and route mileage, were also considered in this design process. The number of departure trips of shuttle buses in each period was last optimized in the frequency-determination model to trade-off passengers’ waiting time and shuttle bus load rate. The main aims of this study were to find the optimal coupling relationship between them by considering the changes in demand and travel time in different periods to guide passengers to choose an optimal ASB route at a nearby station to travel from their working or living places to the airport efficiently and comfortably.

**Figure 2:**
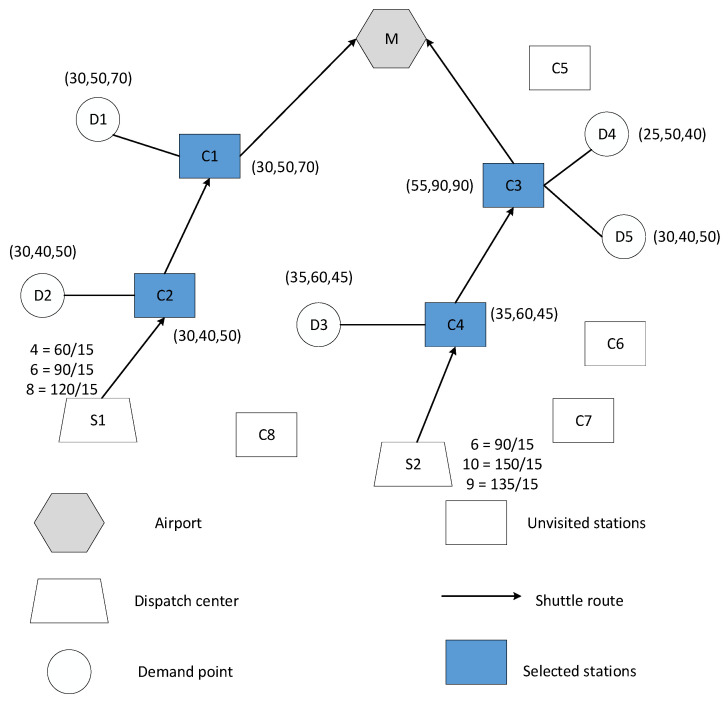
Typical example of the proposed model.

In Figure 2, a small case is shown to illustrate the ASBN model’s specifics further. The ASBN consists of one airport (M), five demand points (D1–D5), eight alternative stations (C1–C8), and two bus dispatch centers (S1 and S2).

In the figure, the numbers of passengers at the demand points in three different periods are indicated by the numbers surrounding the circles. Using demand point D1 as an example, the ridership in the three periods is 30, 50, and 40 persons. The figures surrounding the square are the number of passengers taking the shuttle bus at the selected station in three periods. The optimization process resulted in the allocation of five demand points to four selected stations, depicted as C1 (D1); likewise, additional selected stations could be defined as C2 (D2), C3 (D4, D5), and C4 (D3). Taking C3 (D4, D5) as an example, the number of people boarding at C3 was the sum of those of D4 and D5. Then, two shuttle routes were generated: R1 was described as [S1-C2-C1-M] and R2 was defined as [S2-C4-C3-M]. After the route design was completed, the number of departures per hour could be obtained subject to the constraints of the range of values of departure frequency and full load rate. Taking R1 as an example, the result of dividing the passenger capacity in the first period by the rated capacity of the shuttle bus is the frequency of departures in the first period, i.e., 60/15 = 4 veh/h.

This study proposed a novel multi-objective mixed-integer linear programming (MOMILP) to search for the optimal integrated operation of ASBN locating, routing, and frequency determination in a dynamic environment that simultaneously minimized the total walk or transfer distance between demand points and selected stations, total waiting time at selected stations, and total ride time from stations to airports for all passengers in different periods. Using the following assumptions, the proposed approach was closely aligned with the real-life situation.

- Similarity according to the change characteristics of passenger flow per hour, a day can be divided into several periods, and the characteristics of each period can reflect the overall travel situation;
- An open GIS tool is used to acquire the changes in actual traffic distances and times between such nodes during different periods;
- Big data from mobile signals can be used to obtain the number of passengers at demand points during different periods.

### 4.2. Mathematic Formulation

#### 4.2.1. Notation

The mathematical representations of relevant parameters and variables are condensed in Table 2 to illustrate the proposed model.

#### 4.2.2. Formulation

The objective function (1) targets minimizing the total transfer distance for all passengers during all different periods. The objective function (2) targets the simultaneous minimization of the total ride and waiting times for all passengers during all different periods.

(1) $$min\;f_{1}=\sum_{\forall i\in V}\sum_{\forall j\in C}\sum_{\forall s\in S}h_{ij}\cdot d_{ij}\cdot q_{is}$$

(2) $$minf_{2}=\sum_{\forall j\in C}\sum_{\forall b\in B}y_{j}^{b}\cdot \sum_{\forall i\in I}\sum_{\forall s\in S}h_{ij}\cdot q_{is}\cdot \left[\left(T_{b}^{s}-t_{js}^{b}\right)+\frac{60}{2f_{bs}}\right]$$

Subject to:

(3) $$\;h_{ij}\leq z_{j},\forall i\in V,\forall j\in C$$

(4) $$\sum_{\forall j\in C}h_{ij}=1,\forall i\in V$$

(5) $$\;h_{ij}\cdot d_{ij}\leq G,\forall i\in V,\forall j\in C$$

(6) $$y_{j}^{b}\leq z_{j},\forall b\in B,\forall j\in C$$

(7) $$\sum_{\forall j\in C}y_{j}^{b}\geq 1,\forall b\in B$$

(8) $$\sum_{\forall j\in C}x_{je}^{b}=1,\forall b\in B,\forall e\in M$$

(9) $$\sum_{\forall j\in C}x_{ej}^{b}=0,\forall b\in B,\forall e\in M$$

(10) $$\sum_{\forall j\in C}x_{je}^{b}=0,\forall b\in B,\forall e\in D$$

(11) $$\sum_{\forall j\in C}x_{ej}^{b}=1,\forall b\in B,\forall e\in D\;$$

(12) $$\sum_{\forall e\in C\cup D\cup M}x_{je}^{b}=\sum_{\forall e\in C\cup D\cup M}x_{ej}^{b}=y_{j}^{b},\forall b\in B,\forall j\in C\;$$

(13) $$U_{jb}-U_{eb}\left|C\cup D\cup M\right|\cdot x_{je}^{b}\geq \left|C\cup D\cup M\right|-1\forall b\in B,\forall j,e\in C\;\cup D\cup M$$

(14) $$t_{js}^{b}+t_{je}^{s}+\left(1-x_{je}^{b}\right)H\leq t_{es}^{b},\forall b\in B,\;\forall j,e\in C\cup D\cup M,\;\forall s\in S$$

(15) $$t_{js}^{b}+t_{je}^{s}+\left(1-x_{je}^{b}\right)H\geq t_{es}^{b},\forall b\in B,\;\forall j,e\in C\cup D\cup M,\;\forall s\in S$$

(16) $$q_{js}^{b}+\sum_{\forall i\in V}h_{ie}\cdot q_{is}+\left(1-x_{je}^{b}\right)H\leq q_{es}^{b},\forall b\in B,\forall j,e\in C\;\cup D\cup M,\forall s\in S$$

(17) $$q_{js}^{b}+\sum_{\forall i\in V}h_{ie}\cdot q_{is}+\left(1-x_{je}^{b}\right)H\geq q_{es}^{b},\forall b\in B,\forall j,e\in C\;\cup D\cup M,\forall s\in S$$

(18) $$D_{b}=\sum_{\forall j,e\in C\cup D\cup M}x_{je}^{b}d_{je}\leq D_{max},\forall b\in B$$

(19) $$T_{b}^{s}=\sum_{\forall j,e\in C\cup D\cup M}x_{je}^{b}t_{je}^{s}\geq T_{min}\;\forall b\in B,\forall s\in S$$

(20) $$F_{min}^{s}\leq f_{bs}\leq F_{max}^{s}\forall b\in B,\forall s\in S$$

(21) $$R_{min}^{s}\leq r_{b}^{s}=\frac{\sum_{\forall j\in C}q_{js}^{b}}{Q\cdot f_{bs}}\leq R_{max}^{s},\;\forall b\in B,\forall s\in S.$$

(22) $$\sum_{\forall i\in V}h_{ij}\cdot q_{is}\leq C_{js},\;\forall j\in C,\forall s\in S$$

Constraints (3) and (4) guarantee that a demand point can be assigned to only one selected station. Constraint (5) guarantees that the transfer distance from the selected bus station to each demand point is no more than the maximum acceptable value. Constraint (6) ensures that the shuttle buses cover all selected bus stations. Constraint (7) guarantees that the minimum number of stations chosen visited by each shuttle bus is 1. Constraints (8)-(11) guarantee that each shuttle bus route must regard a dispatch center as its origin and the airport as its eventual destination. Constraint (12) ensures that only one route can serve a selected station. Constraint (13) is used to eliminate the sub-tour. Constraints (14) and (15) compute the reach time of shuttle bus b visiting the present node. Constraints (16) and (17) determine the load capacity of neighboring stations visited by each shuttle bus in each period. Constraints (18) and (19) ensure that the travel mileage and trip time per shuttle bus must satisfy their minimum and maximum values. Constraints (20) and (21) guarantee that each shuttle bus’s frequency and load factor should meet their upper and lower limits in each period. Constraint (22) guarantees that the ridership of taking the bus at bus station j is no more than the capacity of bus station *j* in period *s.*

## 5. Solution Method

**Figure 3:**
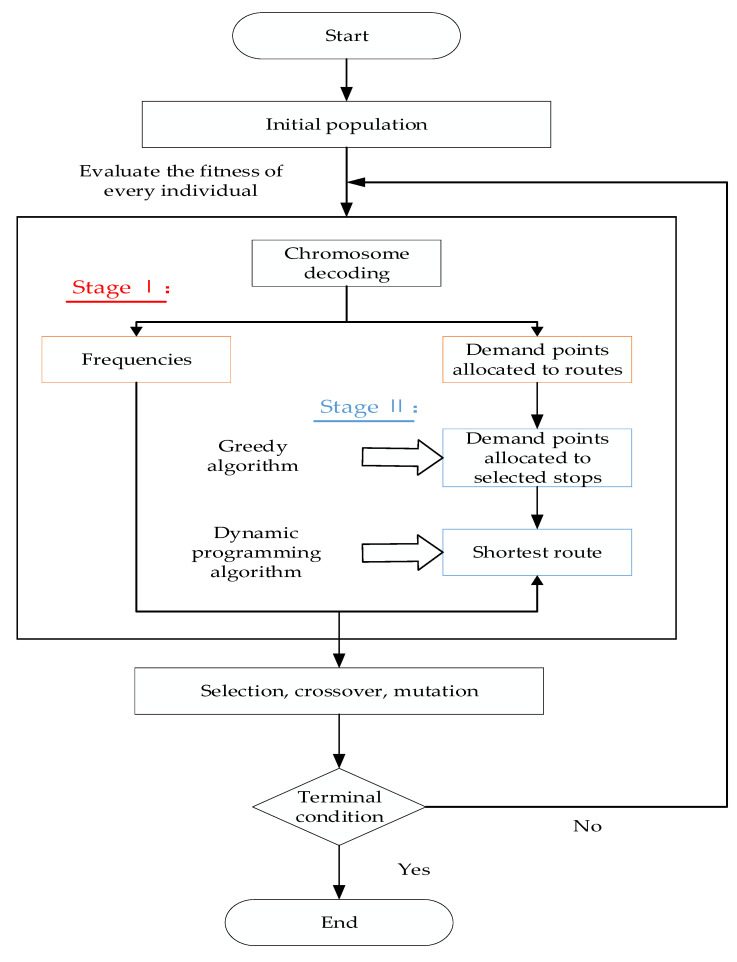
Flowchart of the NSGA-II-based two-phase algorithm.

The proposed ASB model is an expansion of the NP-hard problem associated with the vehicle routing problems (VRP). The exact algorithm is unable to resolve complex issues in a reasonable time. Researchers typically adopt evolutionary algorithms to resolve large-scale problems and complicated optimizations [24,25,27]. To address the proposed problem, a two-phase NSGA-II-based approach is designed in this study. The solution framework of the approach is shown in Figure 3. In the first phase, NSGA-II is applied to assign all demand points to routes and obtain their departure frequencies in multiple periods. In the second phase, the greedy and dynamic programming algorithms are embedded in NSGA-II to determine the selected stations for their demand points and routes from the demand points to the airport. 

### 5.1. Phase I: Allocate Demand Points to Shuttle Bus Routes Using the NSGA-II Algorithm

Utilizing the real number encoding approach, chromosome $X=\left(x_{1},\cdots ,x_{\left|V\right|},x_{\left|V\right|+1},\cdots ,x_{\left|V\right|+\left|S\right|\cdot \left|B\right|}\right)$ denotes a solution to the issue. Chromosome X includes two parts: element $x_{i}\;\left(1\leq i\leq \left|V\right|\right)$ denotes that route $x_{i}$ serves demand point i; element $x_{\left|V\right|+\left(s-1\right)\cdot \left|B\right|+b}\;\left(1\leq b\leq \left|B\right|,1\;\leq s\leq \left|S\right|\right)$ denotes the departure frequencies of each route in different periods [3]. For instance, a solution chromosome including two routes and five demand points at two periods is denoted as X={1, 2, 1, 2, 2;4, 5, 3, 4}, in which (1) demand points 1 and 3 are served by R1, and R2 serves demand points 2, 4, and 5; (2) the two routes’ departure frequencies in the first period are 4 and 5 veh/h, respectively; the two routes’ departure frequencies in the second period are 3 and 4 veh/h, respectively.

In NSGA-II of the first phase, individuals in the initialized population are produced at random. During the decoding process, every demand point is allocated to a route, and each shuttle bus is given its departure frequencies at different periods. After the above operations, the second phase’s dynamic programming algorithm and greedy algorithm are utilized to determine the shuttle bus’s shortest route. Since then, it has been feasible to determine the objective function’s fitness value to establish the fitness value of the objective function and the full shuttle bus operation scheme. By utilizing crossover and mutation operations to swap genes, selected individuals from the parental population are employed to create new individuals. The current population of both new and older individuals is once more sorted and picked to generate offspring. The selection is determined by the rank and crowding distance of the individual. Finding a consistent distribution of solutions along the Pareto front involves using the crowding distance, which is connected to the average length of individuals in the Pareto front. The solutions with the highest crowding distance are picked first if they are in the same Pareto optimum front; otherwise, the lowest-ranked solutions are selected first. The algorithm continues running until the maximum number of iterations are achieved [24].

Furthermore, randomly generated chromosomes may not meet some of the constraints. To assess the quality of chromosomes, penalty functions are used to design fitness functions to extract infeasible solutions from the population during evolution [12].

(23) $$F_{1}=f_{1}+H\cdot \left[\sum_{\forall i\in \in V,\forall j\in C}\max\left\{h_{ij}\cdot d_{ij}-G,0\right\}+\Sigma_{\forall j\in C,\forall s\in S}\max\left\{\sum_{\forall v\in V}\cdot h_{ij}\cdot q_{is}-C_{js},0\right\}\right]+H\cdot \;\sum_{\forall b\in B,\forall s\in S}[\max\{r_{b}^{s}-R_{\max}^{s},0\}\;+\max\{R_{\min}^{s}-r_{b}^{s},0\}]+H\cdot \sum_{\forall b\in B}[\max\{f_{b}-F_{\max},0\}+\max\{F_{\min}-f_{b},0\}]$$

(24) $$F_{2}=f_{2}+H\cdot \sum_{\forall b\in B}[\max\{\sum_{\forall i,j\in C\cup D\cup M}x_{ij}^{b}\cdot d_{ij}-D_{max},0\}+\max\{T_{min}-\sum_{\forall i,j\in C\cup D\cup M}x_{ij}^{b}\cdot t_{ij}^{s},0\}]$$

### 5.2. Phase II: Search the Shortest Shuttle Bus Routes Using Greedy and Dynamic Programming Algorithms

Demand points should be allocated to various routes in the first phase. If each demand point was assigned to a nearby or closest selected station by meeting the station capacity, and the order that the shuttle bus visited selected stations was determined, the complete solution would be acquired. For each demand point in this section, a station is chosen using the minimization of transfer distance principle when its capacity is feasible. In such a case, the dynamic programming algorithm was utilized to search for the shortest path from a dispatch center to an airport to arrange the order of the route’s selected stations. The details of the algorithm are listed in Algorithms 1 [10,28].
**Algorithms 1.** Airport shuttle bus route based on dynamic programming.**Input**N: Number of selected stations, C: Coordinates of selected stations, dispatch center and airport.**Output**Shortest airport shuttle bus route.**Algorithm** **flowchart**  yield selected station collection T and visited station collection V  //calculate the distance between the dispatch center and each selected station and store the  results in array D  **for** t ∈ T **do**    D [1<<(t−1)][t] ← Distance(dispatch center to t)  **end for**  //calculate the distance when all remaining selected stations are visited.  **For** V ∈ [0, 1, 2,…, (2^*N*^ − 1)] **do**    **for** t ∈ T **do**      **if** t has not already visited **then**        **for** previous ∈T **do**          **if** previous has been already visited **then**            D[V|(1<<(t − 1))][t] ← min(D[V][previous]+Distance(previous to t),             D[V|(1<<(t − 1))][t]          **end if**        **end for**      **end if**    **end for**  **end for**  //calculate the distance between the last selected station and airport  min_D ← Infinite   **for** previous ∈T **do**    min_D ← min(D[2*^N^* − 1][previous]+Distance(previous to airport),min_D)  **end for**

## 6. Case Study

### 6.1. Case Description and Data Preparation

**Figure 4:**
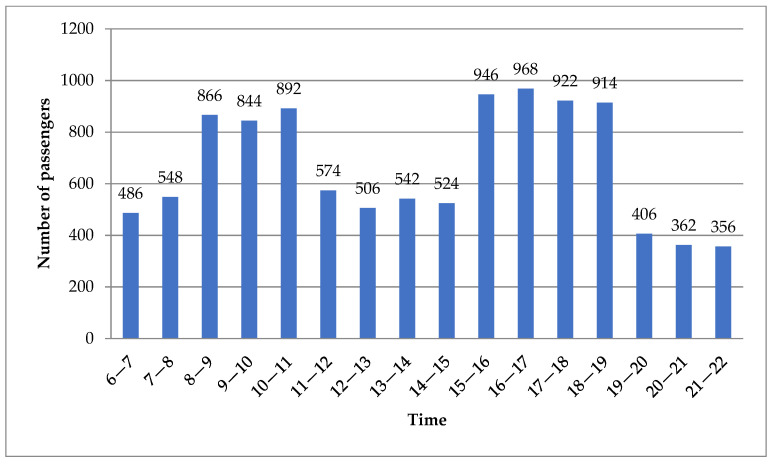
Passenger flow distribution in five periods.

**Figure 5:**
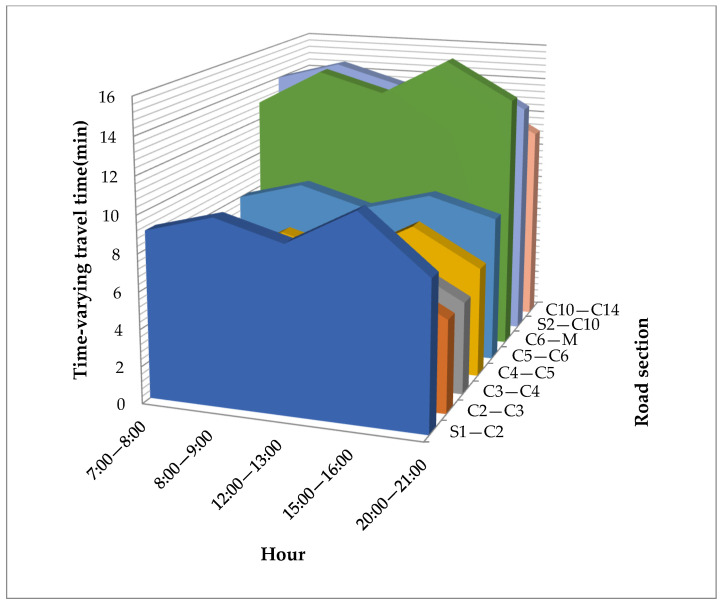
Time-varying travel time of some road sections in five periods.

To illustrate the effectiveness of the proposed optimal model, the Tianjin Airport shuttle bus system was used as an example for analysis. On 7 and 8 May 2022, a total of $3.6\times 10^{8}$ mobile phone data points were gathered to detect 30 demand points and 45 alternative bus stations. After data processing, the total flow of passengers per hour during the daily operating hours was obtained. Furthermore, a time-varying travel time/distance travel matrix between demand points, bus stations, and depots, including 1350 pairs of walking distances and another 2601 pairs of travel distances and times, was extracted from Baidu Map APIs. Although the hourly traffic volume and travel time are not the same, their evolution pattern shows alternating high and low peaks due to the tidal phenomenon of residents’ travel. In general, the whole day can be divided into five different periods, including two peak periods (8:00–11:00 and 15:00–19:00) and three flat periods (6:00–8:00, 11:00–15:00, and 19:00–22:00). Their average passenger flows and travel times in each period can be used to approximate the value of the corresponding hour. Figure 4 and Figure 5 show the time-varying passenger flow and travel time during five different periods, respectively. Table 3 shows the number of passengers at each demand point during five different periods. Hence, the ASBN design result of five periods can be equivalent to those of all different hours in this case.

This case study involves one airport (M), 30 demand points (D1–D30), 45 alternative bus stations (C1–C45), and five dispatch centers (S1–S5) in a dynamic environment. The primary inputs are listed below:

- Number of shuttle bus routes (B): 5;
- Maximum capacity of the shuttle bus (Q): 35 per bus;
- Minimum and maximum load factor ($R_{max}$ and $R_{min}$): 0.4 and 2;
- Maximum transfer distance (G): 5 km;
- Minimum and maximum departure frequencies for five periods ($F_{min}^{t}$ and $F_{max}^{t}$): [2, 3, 2, 4, 2] and [5, 9, 6, 9, 5];
- Parameters of the hybrid algorithm: number of iterations is 500; number of chromosomes is 50; crossover rate is 0.9; mutation rate is 0.1.

### 6.2. Results

**Figure 6:**
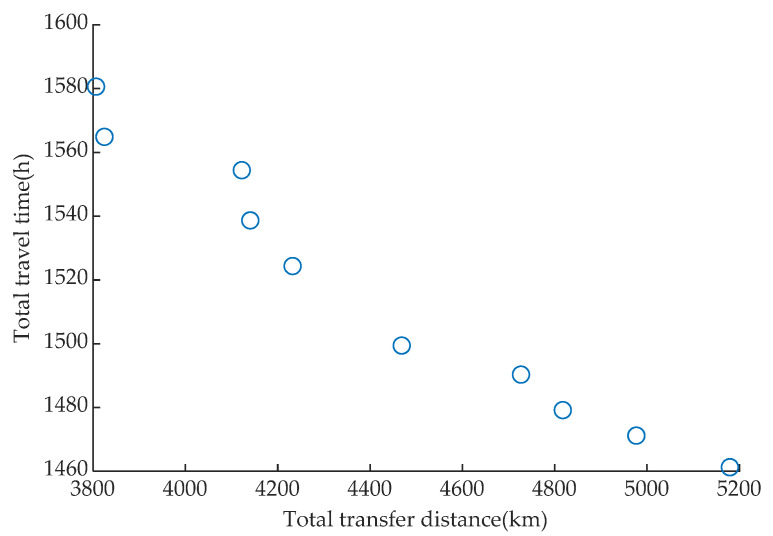
Variation in the relationship between the total transfer distance and total travel time.

The above analysis revealed that the presented model can address two parts of the issue: station choice, passenger guidance, traffic route design, and planning. The maximal and minimal values of objective function 1 were 5179.4 and 3824.8 km, respectively, and those of objective function 2 were 1580.6 and 1464.3 h, respectively. Figure 6 shows the evolution of the relationship between the two objectives. The objective function (2) value was reduced as the objective function (1) grew. The reason was that with the decrease in total transfer distance, vehicles required more miles to cover all selected stations, thereby increasing total travel time.

Table 4 and Table 5 present the routing and scheduling results associated with the Pareto-optimal solution (4727.1, 1490.3). Table 4 specifies the assignment results, which contain the transfer distance. Considering the selected station C2 visited by R1 as an example, passengers D1 and D23 boarded the bus by transferring 0.851 and 1.8495 km, respectively. Table 5 also specifies each route’s routing and scheduling plans, including the load factors and departure frequencies of the five periods. Taking R1 as an example, the frequencies of the five periods were 4, 7, 4, 6, and 4 veh/h, and the load rates of the five periods were 1.0429, 0.9469, 0.9643, 1.2048, and 0.7, respectively.

**Figure 7:**
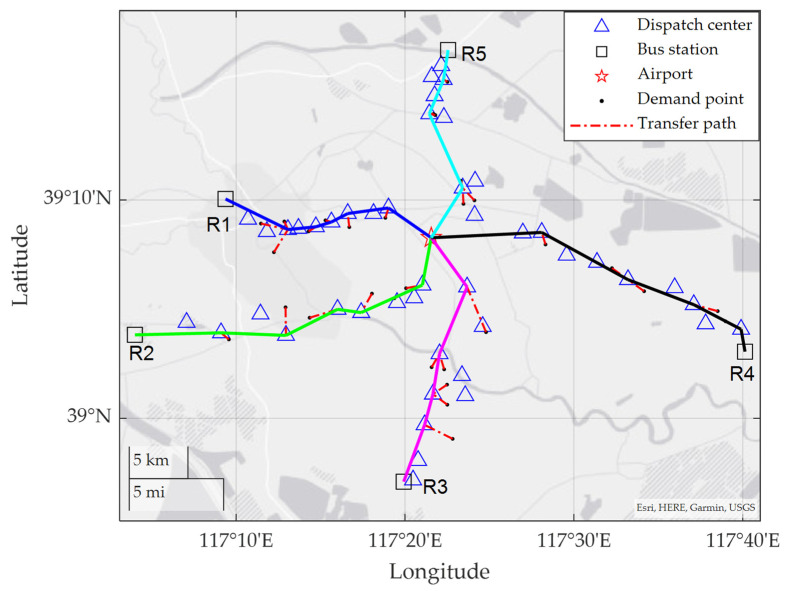
Roadmap of the optimal solution.

Figure 7 shows a map-based bus route plan, and passenger guidance map, where the red star indicates the airport, the blue square indicates a dispatch center, the blue triangle indicates a bus station, and the black dot indicates a demand point. The dark blue solid line denotes R1, the solid green line represents R2, the solid pink line denotes R3, the solid black line denotes R4, and the light blue solid line denotes R5. Except for the ASBN route plan, the transfer paths of passengers are shown by the red dashed lines from the chosen blue triangles to black dots.

**Figure 8:**
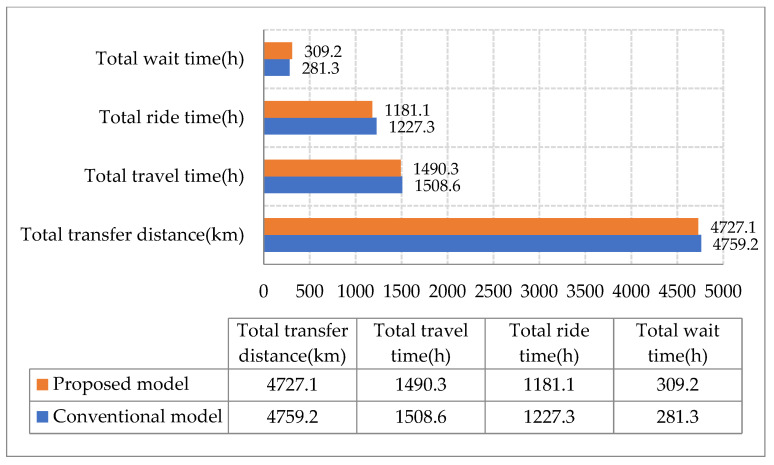
Comparison of the results obtained via the proposed and conventional models.

The proposed and conventional models were compared, as shown in Figure 8.

Compared with the conventional model, the total transfer distance of the proposed model was reduced by 32.1 km. In this research, the total ride time decreased while the total waiting time increased. The objective value in this research was smaller than that of the conventional model, as the increase in the former was less than the decrease in the latter. This was because the study took the route design and departure frequency into account to seek global optimization, preventing the local optimum of the conventional approach, in which the route design was decided first, and then the departure frequency was computed.

### 6.3. Comparative and Sensitivity Analyses

Sensitivity analyses were reformed to examine the effects of the number of routes, station capacity, and load factor on the model performance. Table 6 summarizes the effect of the number of routes.

Based on the results listed in Table 6, the following patterns were revealed:

(1) The growth in the number of routes resulted in some erroneous mileage and time, which increased the total route mileage because routes start at the dispatch center and end at the airport. Nevertheless, as the number of routes expanded, fewer stations were visited by each route, which decreased the passengers’ total ride time.
(2) The ridership at demand points visited by each route declined as the number of routes increased, resulting in a decreased departure frequency. In this scenario, all passengers’ waiting times steadily increased. However, the total frequency increased, which led to a decrease in the mean load factor.
(3) The total transfer distance fluctuated erratically with the number of routes raised.

The station capacity effect on the proposed model performance is shown in Table 7, where ΔC denotes the increment in station capacity.

The following correlations can be observed from the data in Table 7:

(1) Due to the increased station capacity, there was a reduction in the number of selected stations of each route, which resulted in a reduction in the total route mileage and the total ride time of passengers. However, as the number of selected stations decreased, the total transfer distance increased because the station nearest to the demand point may not have been selected.
(2) As the station capacity increases, some stations may be assigned to different routes, which leads to changes in the total frequency and mean load factor. The total frequency changed irregularly, as did the total waiting time. Although, as station capacity increased, the total waiting time varied irregularly, the sum of the total ride time and the total waiting time gradually decreased, so the total travel time decreased.

The load factor effect on the proposed model performance is described in Table 8, where ΔR is the load factor increment.

The following is a summary of the key findings:

(1) As the load factor increased, the total frequency decreased, which increased the total waiting time. At the same time, the reduction in total frequency led to a decrease in the total route mileage and total trip time.
(2) As the number of selected stations was not affected by the load rate, the total ride time remained essentially the same. The change in the total waiting time was more significant than the change in the total ride time; therefore, the trend in total travel time was associated with an increase in total passengers’ waiting time.
(3) The total transfer distance changed irregularly with the increased load factor.

**Figure 9:**
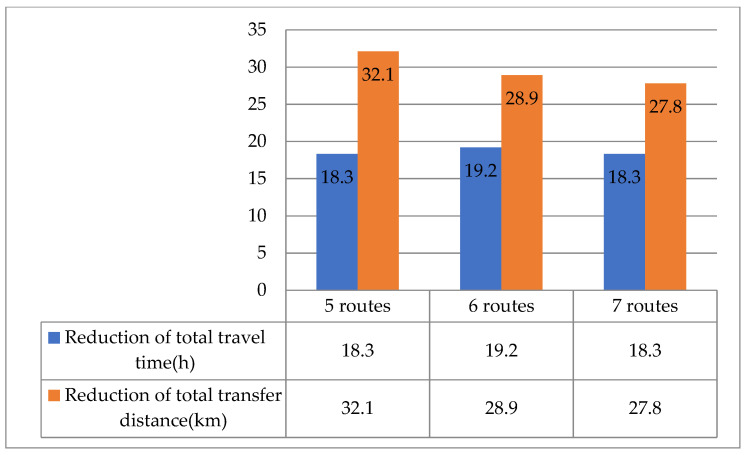
Comparative analysis of the conventional and proposed models for various numbers of routes.

**Figure 10:**
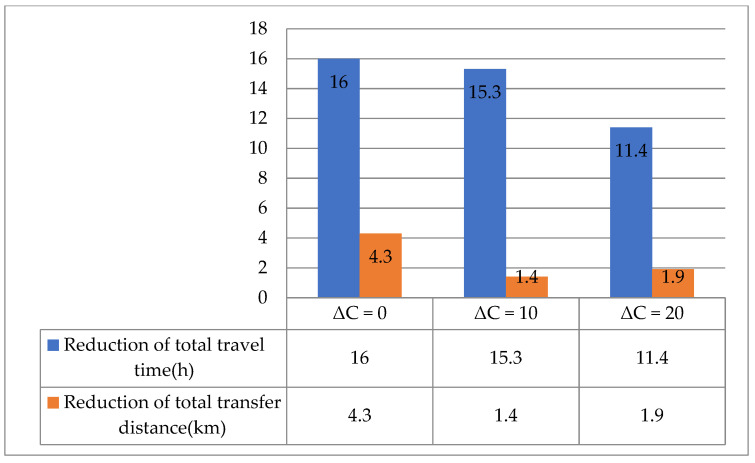
Comparative analysis of the conventional and proposed models for various station capacities.

**Figure 11:**
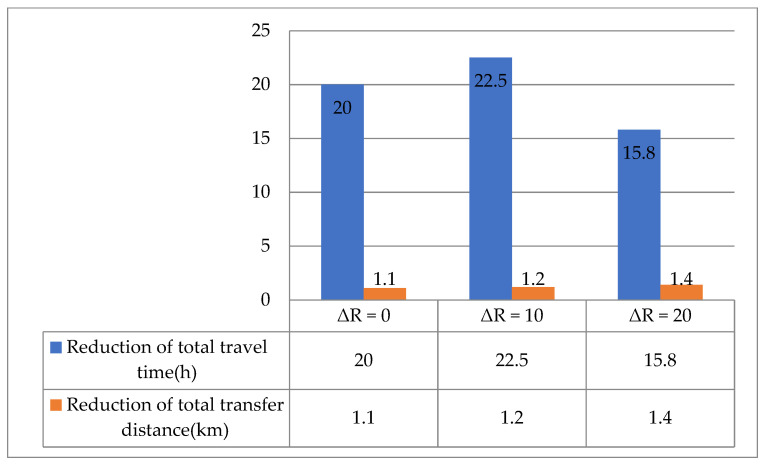
Comparative analysis of the conventional and proposed models for various factors.

In addition, Figure 9, Figure 10 and Figure 11 illustrate the difference in model performances of the conventional and proposed models under different scenarios. The total travel time and transfer distance of the proposed model were reduced compared to the conventional model under all scenarios. The differences in model performance are consistent with the patterns in Figure 8, further verifying the proposed model’s feasibility.

## 7. Conclusions

This research proposed a comprehensive model for a dynamic ASB of a low-carbon and green travel mode to coordinate station selection, route design of shuttle buses, and frequency setting simultaneously to balance the operation efficiency according to time-varying demand and travel time. A two-phase multi-objective non-dominated sorting genetic (NSGA-II) algorithm was further designed to solve this NP-hard problem. Combined with the dynamic programming search method, the proposed algorithm was used to acquire the allocation of demand points to different routes and to determine the frequency of departure in the first phase. In the second phase, the greedy and dynamic programming algorithms were incorporated into the NSGA-II algorithm to assign demand points to selected stations and to compute the shortest route for the shuttle bus. The results of an example study indicated that the total passenger travel time of the presented model was markedly reduced by 1.21% compared with the conventional model. The primary cause was that the conventional model neglected to seek global optimization in all operational periods, instead only considering the optimal route and departure frequency for a single period. This study investigated the variance in solution performance between the conventional and presented models in five routes to assess the validity of the proposed model. The proposed model’s transfer distance shrank compared to the conventional model, and its total travel time was shorter. The findings indicated that the proposed approach might optimize station selection, shuttle transit routing, and frequency determination. Additionally, sensitivity tests were performed to explore how the load factor constraint, station capacity, and the number of routes affected the model’s performance.

However, this study had several limitations. First, it envisaged that only one selected station could be designated at each demand point, which ignored the concept that when the ridership at the demand point was more than the capacity of the route, the travel demand would be separated. Each set of passengers could choose diverse stations to board the shuttle bus. Secondly, this study was based on a hypothesis of stable travel demand, which did not consider the stochastic nature and uncertainty of travel demand. In real life, the travel demand depends on real-time traffic conditions, weather, time urgency for passengers, etc. Therefore, considering the stochastic nature and uncertainty of travel demand would make the model more realistic. Thirdly, this study assumed that the dispatch centers’ locations were stable, ignoring the balance between the shuttle bus route design and the dispatch centers’ locations. A follow-up study could be a proposed model extension to the problem containing splitting demand with stochastic nature and uncertainty and the interactive process of designing shuttle bus routes and the locations of dispatch centers.

## Figures and Tables

**Table 1:** State-of-the-art ASBN/CBN integrated optimization models.

Demand Pattern	Objective	Solution Method	RD	FD	SS	TVD	Reference
Static	The number of passenger transfers	Hybrid artificial bee colony algorithm.	Yes	Yes	No	No	[8]
Static	The sum of all passengers’ walking and traveling time.	A two-phase genetic algorithm combining the Dijkstra search method.	Yes	Yes	Yes	No	[12]
Static	The total time spent by users; the operational cost	The multi-objective bi-level approach based on global-best harmony search.	Yes	Yes	No	No	[13]
Static	The passenger cost; the unmet demand	Differential evolution approach.	Yes	Yes	No	No	[15]
Static	The total waiting time of all passengers.	Simulated annealing.	Yes	Yes	No	No	[17]
Static	Passenger ride time and operator costs	Corridor generation algorithm.	Yes	Yes	No	No	[18]
Static	The time difference between buses that cannot be reached synchronously	A heuristic algorithm	Yes	Yes	No	No	[19]
Static	The total operation cost; the total passenger travel cost.	Iterative layered optimization algorithm with forward-passing and backpropagation based on Lagrangian duality	Yes	Yes	Yes	No	[20]
Static	Operating costs; total travel times.	An outer approximation method	Yes	Yes	Yes	No	[21]
Dynamic	The weighted overall passenger’s waiting time; passengers’ ride time; operation costs.	Improved Lagrangian relaxation algorithm.	Yes	Yes	Yes	No	[22]
Dynamic	The amount of time passengers wait for shuttles; the shuttle energy consumption.	A discrete-event simulator	Yes	Yes	No	Yes	[23]
Dynamic	Total transfer distance of all passengers; total ride and waiting time of all passengers.	Two-phase NSGA-II-based approach.	Yes	Yes	Yes	Yes	This paper

Note: RD is route design, FD is frequency determination, SS is station selection, and TVD is time-varying demand.

**Table 2:** Parameters and variables in the ASB model.

Indices:	
i	Demand point index
j,e	Shuttle bus node (dispatch center, station, and airport) index
b	Shuttle bus route index
s	Period index
**Sets:**	
V	Set of demand points
B	Set of shuttle bus routes
C	Set of alternative stations
D	Set of dispatch centers
M	Set of airports
S	Set of periods
**Parameters:**	
$q_{is}$	Ridership at demand point i in period s; ∀i∈V, ∀s∈S
Q	Maximum capacity of the shuttle bus
$F_{max}^{s}$	Maximum departure frequency in period s; ∀s∈S
$F_{min}^{s}$	Minimum departure frequency in period s; ∀s∈S
$r_{b}^{s}$	Load rate of route b in period s; ∀b∈B, ∀s∈S
$R_{max}^{s}$	Maximum load rate in period s; ∀s∈S
$R_{min}^{s}$	Minimum load rate in period s; ∀s∈S
$D_{max}$	Maximum mileage of shuttle bus route
$T_{min}$	Minimum travel time of shuttle bus route
$D_{b}$	The total mileage of route b visiting all shuttle bus stations; ∀b∈B
$T_{b}^{s}$	The total travel time of route b visiting all shuttle bus stations in period s; ∀b∈B,∀s∈S
G	Maximum transfer distance
$d_{ij}$	Map-based distance from the airport, bus stations, and dispatch centers to demand points i and j,∀i,j∈V∪M∪D∪C
$t_{je}^{s}$	Map-based time from bus stations and dispatch centers to airport j and e in period s;∀j,e∈M∪D∪C,∀s∈S
$C_{js}$	The capacity of bus station j in period s; ∀j∈C,∀s∈S
H	A large constant
**Decision variables:**	
$z_{j}$	Whether the alternative station j is selected as a shuttle bus station; ∀j∈C
$h_{ij}$	Whether the demand point i is allocated to a shuttle bus station j; ∀i∈V,∀j∈C
$x_{je}^{b}$	Whether the shuttle bus station j precedes shuttle bus station e on route k; ∀b∈B,∀j,e∈D∪M∪C
$f_{bs}$	Frequency of route b in period s; ∀b∈B, ∀s∈S
$y_{j}^{b}$	Whether the shuttle bus station j is served by route b;∀j∈D∪M∪C,∀b∈B
$t_{js}^{b}$	The time of route b arriving at the shuttle bus station j in period s; ∀j∈D∪M∪C,∀b∈B,∀s∈S
$q_{js}^{b}$	Ridership at the shuttle bus node j allocated to route b in period s;∀j∈C,∀b∈B,∀s∈S
$U_{jb}$	An auxiliary (real) variable for sub-tour elimination constraint in route of shuttle bus b;∀j∈D∪M∪C,∀b∈B

**Table 3:** Data on all demand points.

No.	$q_{i1}$	$q_{i2}$	$q_{i3}$	$q_{i4}$	$q_{i5}$
D1	23	36	21	40	15
D2	22	35	20	38	15
D3	18	29	17	31	12
D4	27	43	25	47	18
D5	22	35	20	38	15
D6	16	25	15	28	11
D7	24	38	22	42	16
D8	13	21	12	23	9
D9	19	30	18	33	13
D10	13	21	12	23	9
D11	20	32	19	35	13
D12	15	24	14	26	10
D13	16	25	15	28	11
D14	20	32	19	35	13
D15	15	24	14	26	10
D16	19	30	18	33	13
D17	12	19	11	21	8
D18	13	21	12	23	9
D19	11	17	10	19	7
D20	15	24	14	26	10
D21	24	38	22	42	16
D22	11	17	10	19	7
D23	18	29	17	31	12
D24	12	19	11	21	8
D25	18	29	17	31	12
D26	23	36	21	40	15
D27	26	41	24	45	17
D28	25	40	23	44	17
D29	12	19	11	21	8
D30	24	38	22	42	16

**Table 4:** Assignment results for all demand points to selected stations visited by routes.

Demand Point	Selected Stations	Assigned Route	Transfer Distance (km)
D1	C2	R1	0.8
D23			1.8
D2	C3		0.7
D5	C4		0.7
D3	C5		0.5
D4	C6		1.2
D6	C7		1.1
D8	C10	R2	0.9
D7	C12		4.4
D9			2.5
D10	C13		1.9
D12	C15		0.4
D11	C16		1.1
D13	C20	R3	2.7
D15	C22		1.4
D17			1.3
D16	C23		4.2
D14	C24		1.5
D18			1.4
D22	C29	R4	1.4
D19	C30		2.1
D20			1.7
D21	C33		1.7
D24	C36		1.1
D28	C41	R5	0.6
D30			1.3
D29	C42		1.3
D25	C43		0.4
D26	C44		0.4
D27			0.5

**Table 5:** Routing and scheduling plans.

Route	Sequence of Stations Visited by Routes	$f_{B1}$	$f_{B2}$	$f_{B3}$	$f_{B4}$	$f_{B5}$	$r_{B}^{1}$	$r_{B}^{2}$	$r_{B}^{3}$	$r_{B}^{4}$	$r_{B}^{5}$	Route Mileage (km)	Travel Time (h)
R1	S1-C2-C3-C4-C5-C6-M	4	7	4	6	4	1.0	0.9	1.0	1.2	0.7	19.2	0.6
R2	S2-C10-C14-C12-C17-M	4	7	5	7	4	0.7	0. 7	0.6	0.7	0.5	29.5	0.9
R3	S3-C20-C24-C22-M	4	6	3	7	4	0.7	0.6	0.8	0.7	0.5	22.7	0.7
R4	S4-C28-C29-C36-M	5	8	4	8	3	0.5	0.5	0.5	0.5	0.5	29.7	0.9
R5	S5-C40-C39-C42-M	3	7	5	7	4	0.9	0.8	0.7	0.9	0.6	19.3	0.6

**Table 6:** Effect of the number of routes on the proposed model performance.

Scenario	Total Travel Time (h)	Total Riding Time (h)	Total Waiting Time (h)	Total Transfer Distance (km)	Mean Load Factor	Total Frequency	Total Route Mileage (km)	Total Trip Time (h)
5 routes	1490.3	1181.1	309.2	4727.1	0.7067	130	601.8	18.5
6 routes	1470.2	1155.7	314.5	4732.4	0.6851	134	695.5	21.3
7 routes	1448.6	1129.3	319.3	4729.8	0.6621	139	780.5	23.8

**Table 7:** Influence of station capacity on the proposed model performance.

Scenario	Total Travel Time (h)	Total Riding Time (h)	Total Waiting Time (h)	Total Transfer Distance (km)	Mean Load Factor	Total frequency	Total Route Mileage (km)	Total Trip Time (h)
ΔC = 0	1490.3	1181.1	309.2	4791.1	0.7067	130	601.8	18.5
ΔC = 10	1484.3	1176.5	307.8	4798.2	0.6902	132	591.4	18.3
ΔC = 20	1478.1	1169.2	308.9	4809.3	0.6959	131	635.2	19.4

**Table 8:** Load factor effect on the proposed model performance.

Scenario	Total Travel Time (h)	Total Riding Time (h)	Total Waiting Time (h)	Total Transfer Distance (km)	Mean Load Factor	Total Frequency	Total Route Mileage (km)	Total Trip Time (h)
Δ*R* = 0	1490.3	1171.1	319.2	4727.1	0.7067	130	601.8	18.5
Δ*R* = 0.1	1513.7	1175.1	338.6	4731.2	0.7223	127	556.8	17.2
Δ*R* = 0.2	1523.6	1173.2	350.4	4729.4	0.7468	123	514.6	16.3

## Data Availability

Some or all data, models, or code generated or used during the study are available from the corresponding author by request.
